# Baseline Investigation of Non-Wild-Type Bacterial Indicators and Antibiotic Resistance Genes in Urban Wastewater from Patras, Greece

**DOI:** 10.3390/microorganisms14081714

**Published:** 2026-08-04

**Authors:** Zoi Anastopoulou, Konstantina Charalambous, Angelos Padouvas, Rafail Fokas, Kalypso-Angeliki Koukouvini, Maria Athanasiou, Nikolaos Giormezis, Despoina Gkentzi, Apostolos Vantarakis

**Affiliations:** 1 Laboratory of Public Health, Epidemiology and Quality of Life, School of Medicine, University of Patras, 26504 Patras, Greece; zoi.anastopoulou@gmail.com (Z.A.); kwnstantina.ch2002@gmail.com (K.C.); angelosemman@gmail.com (A.P.); fokasrafael@gmail.com (R.F.); k.koukouvini@gmail.com (K.-A.K.); mariaathanasioupatra@gmail.com (M.A.); 2 Department of Microbiology, School of Medicine, University of Patras, 26504 Patras, Greece; giormenik@upatras.gr; 3 Department of Paediatrics, School of Medicine, University of Patras, 26504 Patras, Greece; gkentzid@upatras.gr

**Keywords:** antimicrobial resistance genes, wastewater surveillance, non-wild-type phenotypes, antimicrobial susceptibility testing, *Escherichia coli*, *Pseudomonas aeruginosa*, *Enterococcus* spp., real-time PCR

## Abstract

Antimicrobial resistance (AMR) is a major public health concern, and wastewater monitoring can complement clinical surveillance by capturing resistance signals at the population level. This exploratory study assessed selected antibiotic-resistant bacteria and antimicrobial resistance genes (ARGs) in untreated influent wastewater from the municipal wastewater treatment plant of Patras, Greece. *Escherichia coli*, *Pseudomonas aeruginosa*, and *Enterococcus* spp. were isolated using culture-based methods, tested for antimicrobial susceptibility by disk diffusion and Etest according to EUCAST epidemiological cut-off values, and screened by real-time PCR for *intI1*, *sul1*, *qnrS1*, *bla*TEM, *bla*VIM, *vanA*, and *ermB*. Among 16 *E. coli* isolates, non-wild-type (non-WT) profiles were detected on 14/16 for meropenem and 15/16 for ciprofloxacin, whereas only 1/16 was non-WT for ampicillin. All 13 *P. aeruginosa* isolates were wild type (WT) for meropenem but non-WT for ciprofloxacin, while all 17 *Enterococcus* spp. isolates were WT for vancomycin and ampicillin. Molecular screening showed that *bla*TEM was the most frequently detected gene in *E. coli*, while intI1 and sul1 were detected in subsets of *P. aeruginosa* isolates. No targeted ARGs were detected in *Enterococcus* spp. These findings provide preliminary, site-specific baseline information on selected AMR phenotypes and genetic determinants in wastewater-derived bacterial isolates from Patras.

## 1. Introduction

Epidemiological surveillance is a fundamental function of Public Health, involving continuous and systematic collection, integration, analysis, interpretation, and timely dissemination of health-related data to support evidence-based public health action. Effective surveillance systems are essential for identifying priorities, assessing risks, monitoring epidemiological trends, and evaluating the impact of prevention and control measures at the population level. In this context, environmental monitoring has become increasingly important, as it provides insight into long-term ecological and microbial trends, supports policy evaluation, and contributes to the generation of new research hypotheses [1].

Wastewater-based epidemiology (WBE) has emerged as an innovative and complementary approach to conventional clinical surveillance, offering population-level information on the circulation of pathogens and other health-related biomarkers within a community. Through the analysis of raw wastewater samples, WBE enables the detection of biological markers, including DNA and RNA from microorganisms shed by infected or colonized individuals. Unlike clinical surveillance, which relies on individual testing, wastewater surveillance provides aggregated data from the population served by a sewerage network, without identifying individuals [2]. Initially applied in 2005 for monitoring illicit drug use, WBE has since evolved into a valuable public health tool capable of tracking infectious diseases, pharmaceuticals, antibiotics, and antimicrobial resistance indicators [3]. Its relevance was strongly demonstrated during the COVID-19 pandemic, when wastewater monitoring provided early information on viral circulation at the community level, often before increases in clinically reported cases [4].

Urban wastewater is a complex matrix composed of domestic, commercial, industrial, agricultural, and stormwater inputs. Domestic wastewater, in particular, contains human excreta and represents a major source of microorganisms entering sewerage systems [5]. Wastewater may contain a broad range of bacteria, viruses, protozoa, and parasites, making it an important substrate for studying microbial circulation and environmental dissemination. Among these microorganisms, bacteria represent one of the most diverse groups of human pathogens detected in wastewater. Several bacterial species colonize the human intestine and are excreted in faeces; while many are commensal or beneficial, others are pathogenic and may contribute to the microbial burden of wastewater. Important bacterial pathogens or opportunistic pathogens associated with wastewater include *Salmonella* spp., *Escherichia* spp., *Shigella* spp., *Yersinia* spp., *Klebsiella* spp., *Leptospira* spp., *Vibrio cholerae*, *Aeromonas hydrophila*, *Legionella pneumophila*, and *Pseudomonas* spp. Some of these organisms, such as *L. pneumophila*, *Mycobacterium avium*, *Pseudomonas aeruginosa*, and *A. hydrophila*, are primarily environmental opportunistic pathogens that may cause disease in individuals [6].

Τreatment plants are of particular interest for Antimicrobial resistance (AMR) surveillance, as they receive antibiotics, Antibiotic-Resistant Bacteria (ARBs), and antimicrobial resistance genes (ARGs) from human, animal, healthcare, and environmental sources. A substantial proportion of antibiotics may be excreted in active forms and enter wastewater systems, where they may exert selective pressure and contribute to the persistence and dissemination of resistant bacteria and ARGs [7]. Through the discharge of treated effluents, resistant microorganisms and resistance determinants may re-enter aquatic and terrestrial ecosystems, reinforcing the environmental dimension of AMR transmission [8]. Recent research conducted in Patras further supports the value of WBE beyond viral monitoring, demonstrating its applicability for detecting pharmaceuticals and antibiotics and for generating information relevant to public health and the environmental dissemination of AMR [9].

AMR occurs when microorganisms, including bacteria, viruses, fungi, and parasites, adapt and grow in the presence of antimicrobial agents that were previously effective against them. Infections caused by resistant microorganisms are associated with increased morbidity, prolonged hospitalization, higher healthcare costs, use of second-line or more toxic drugs, and increased treatment failure [10]. AMR is driven by several interconnected factors, including inappropriate and excessive antimicrobial use, limited access to clean water, sanitation and hygiene, inadequate infection prevention and control measures, insufficient awareness, and weaknesses in legislation and implementation [7]. At the molecular level, AMR is associated with the presence and expression of ARGs, which may be maintained and disseminated through selection pressure, genetic mutations, and horizontal gene transfer [11,12]. Mobile genetic elements, including plasmids, integrons, and transposons, further facilitate the spread of resistance among bacterial species [8]. Key resistance mechanisms include enzymatic inactivation of antibiotics, modification of target sites, reduced intracellular drug accumulation through efflux pumps, and biofilm formation, which can reduce antimicrobial efficacy [13].

Within wastewater-based AMR surveillance, *Escherichia coli* (*E.coli*), *Pseudomonas aeruginosa* (*P. aeruginosa*), and *Enterococcus* spp. are of relevance. *E. coli* is a Gram-negative bacillus associated with diarrhoeal disease, urinary tract infections, bacteraemia, pneumonia, and intra-abdominal infections [14]. Although intrinsically susceptible to many clinically relevant antimicrobial agents, *E. coli* has a high capacity to acquire and accumulate resistance genes, mainly through horizontal gene transfer [15]. It is also widely used as an indicator of faecal contamination in environmental waters and as a useful organism for AMR monitoring, given its role as both donor and recipient of ARGs [16].

*P. aeruginosa* is a Gram-negative, aerobic, non-spore-forming opportunistic pathogen capable of causing a wide range of infections, particularly in immunocompromised individuals and patients with cystic fibrosis [17]. Its clinical importance is strongly linked to its intrinsic and acquired resistance to multiple antibiotics, its ability to form biofilms, and its capacity to develop multidrug-resistant phenotypes. These characteristics limit therapeutic options and make *P. aeruginosa* infections difficult to treat [18].

*Enterococci* are Gram-positive facultative anaerobic cocci commonly found in human faeces and the environment. They are important causes of healthcare-associated infections, including urinary tract infections, bacteraemia, infective endocarditis, and, less frequently, intra-abdominal infections and meningitis [14]. Due to their persistence in the environment and association with faecal contamination, enterococci are widely used as indicators of water quality and faecal pollution [19,20]. Their clinical significance is closely related to their intrinsic resistance to several antimicrobial agents and their ability to acquire and transfer resistance determinants through mobile genetic elements [21].

The global public health importance of AMR is substantial. According to the World Health Organization (WHO), more than 1.27 million deaths globally are directly attributable to infections caused by antibiotic-resistant bacteria worldwide [22]. The spread of resistant microorganisms reduces available treatment options, compromises the effectiveness of essential medical procedures that depend on antimicrobial prophylaxis or therapy, and increases the burden on healthcare systems. Because AMR arises and circulates across humans, animals, and the environment, its monitoring requires a One Health approach that recognizes the interconnectedness of these sectors [7,22].

WBE provides a unique opportunity to integrate the surveillance of viral pathogens and AMR indicators within a single population-level monitoring framework. Wastewater analysis can be used to detect respiratory viruses, while simultaneously assessing the occurrence of ARBs and ARGs. Although viruses and bacteria differ in their biology and transmission pathways, their co-occurrence in wastewater allows the combined surveillance of infectious disease trends and resistance dynamics. Such an integrated approach can support early warning, improve understanding of community-level health risks, and strengthen evidence-based decision-making in public health [22,23].

The aim of the present study was to investigate non wild type (non-WT) bacterial indicators and ARGs from the inlet wastewater using microbiological and molecular techniques. The present study was designed as an exploratory, site-specific baseline investigation of selected bacterial indicators and antimicrobial resistance determinants in untreated municipal wastewater from Patras, Greece. Specifically, it combined culture-based antimicrobial susceptibility testing of wastewater-derived *E. coli*, *P. aeruginosa*, and *Enterococcus* spp. isolates with targeted real-time PCR screening for a restricted panel of ARGs. The study was not intended to characterize the complete wastewater resistome, determine ARG host range beyond the cultured isolates, investigate horizontal gene transfer mechanisms, or perform quantitative risk assessment. Rather, its objective was to generate preliminary local data that may inform the design of broader longitudinal AMR monitoring studies in Patras wastewater catchment.

## 2. Materials and Methods

### 2.1. Study Design and Sampling Site

The study was conducted to investigate the occurrence of non-WT bacterial indicators and selected antimicrobial resistance genes in urban wastewater. Wastewater samples were collected from the inlet of the municipal wastewater treatment plant of Patras, Greece, prior to treatment. The facility serves an estimated population of approximately 168,000 inhabitants. Sampling was carried out by the technical personnel of the wastewater treatment plant using 24-h composite sampling. Following collection, the samples were transported to the laboratory in a portable cooler at approximately 5–8 °C, stored under refrigeration at 5 °C, and analysed no later than the following day. This sampling strategy was applied to characterize the microbial load entering the treatment facility and to obtain site-specific information on bacterial contamination and antimicrobial resistance indicators in untreated influent wastewater.

### 2.2. Bacterial Isolation and Identification

For bacterial analysis, 10 mL of diluted wastewater samples (10^−3^ and 10^−4^ dilutions) were filtered through 45-mm membrane filters, which were subsequently placed on selective culture media for the isolation of target bacterial groups. *E. coli* was cultured on Chromocult Coliform Agar and incubated at 37 °C for 24 h, in accordance with EN ISO 9308-1 [24]. *Enterococcus* spp. was cultured on Slanetz and Bartley Agar and incubated at 36 ± 2 °C for 48 h, in accordance with EN ISO 7899-2 [25], while *P. aeruginosa* was cultured on Pseudomonas Agar Base (CN-supplemented) and incubated at 36 ± 2 °C for 48 h, in accordance with EN ISO 16266 [26].

Identification of *E. coli* was based on characteristic colony morphology and coloration on the respective selective medium, with blue colonies considered indicative of *E. coli.*, *P. aeruginosa* colonies were identified presumptively on the basis of green fluorescence under UV light (360 ± 20 nm), without further biochemical confirmation. Presumptive *Enterococcus* spp. colonies were confirmed by transferring the membrane filter onto pre-warmed (44 °C) Bile Aesculin Azide Agar and incubating for 2 h, following the rapid confirmation protocol of EN ISO 7899-2, the appearance of a black/brown discoloration in the surrounding medium was recorded as a positive reaction.

One representative colony per bacteria per sample was selected using a sterile microbiological loop and subcultured in Tryptone Soy Broth (TSB) for 24 h. From each culture, a bacterial suspension was prepared and adjusted spectrophotometrically to a turbidity of 0.5 McFarland (corresponding to approximately 1–2 × 10^8^ CFU/mL), in accordance with the EUCAST guidelines (v.15.0). Within 15 min of preparation, each suspension was inoculated onto the entire surface of Mueller–Hinton Agar plates using a sterile cotton swab to obtain confluent (lawn) growth. Antimicrobial susceptibility was assessed by both the disk diffusion method and gradient strip testing (Etest), antimicrobial disks and Etest strips were applied within 15 min of inoculation, and plates were incubated at 35 ± 1 °C in air for 18 ± 2 h (24 h for vancomycin testing of *Enterococcus* spp.). Inhibition zone diameters and minimum inhibitory concentration (MIC) were interpreted on the basis of the EUCAST epidemiological cut-off values (ECOFFs, v.15.0), and isolates were classified as WT or non-WT accordingly.

### 2.3. Antimicrobial Susceptibility Testing

Antimicrobial susceptibility testing was performed using the disk diffusion and Etest methods, with results interpreted on the basis of the EUCAST ECOFFs, classifying isolates as WT or non-WT. The Etest method was used to determine the MIC of the selected antibiotics, and the results were interpreted against the corresponding MIC-based ECOFFs (mg/L), whereas disk diffusion was used to measure inhibition zone diameters, which were interpreted against the corresponding zone-diameter ECOFFs (mm). Non-WT profiles were considered indicative of reduced susceptibility and the possible presence of acquired resistance mechanisms. Bacterial culture assays were performed in duplicate for each sample. EUCAST ECOFFs values are mentioned in Table 1.

Two points regarding the *Enterococcus* spp. analysis should be noted. The isolates were not identified to the species level (e.g., *E. faecalis* versus *E. faecium*) as all *Enterococcus* spp. isolates in this study were classified as WT, the classification was unambiguous regardless of species, and species-level identification was therefore not required for the interpretation of the results and was not pursued. Moreover, the vancomycin ECOFF 4 mg/L is identical for both species and was thus unaffected by this consideration.

### 2.4. Molecular Detection of Antimicrobial Resistance Genes

For molecular analysis, well-isolated single colonies from each bacterial culture were inoculated into (TSB) and incubated for 24 h under agitation to obtain sufficient biomass. Total genomic DNA was extracted using the NucleoSpin Microbial DNA kit (Macherey-Nagel, Düren, Germany) according to the manufacturer’s instructions. The presence of antimicrobial resistance genes was assessed by real-time PCR. The targets investigated were *intl1*, *sul1*, *qnrS1*, *bla*TEM, *bla*VIM, *ermB* and *vanA*, selected to represent genetic determinants associated with mobile genetic elements (*intl1*), sulfonamide resistance (*sul1*), quinolone resistance (*qnrS1*), β-lactam resistance (*bla*TEM), carbapenem resistance (*bla*VIM), macrolide resistance (*ermB*) and vancomycin resistance (*vanA*). In addition, a housekeeping reference gene was included for each organism to confirm successful DNA extraction, template integrity and bacterial identity: the *gyrA* gene for *P. aeruginosa* and the 16S rRNA gene (16S rDNA) for *E. coli* and *Enterococcus* spp. These targets served as internal amplification and process controls and were not used for the assessment of antimicrobial resistance.

Following determination of the amount of extracted DNA using Qubit Fluorometer, and detection of ARGs using real-time PCR, Aria MX (Agilent Technologies, Santa Clara, CA, USA) and Luna Universal Master Mix SYBR green (New England Biolabs, Ipswich, MA, USA) using a specific set of primers for each gene. The primer sequences and annealing temperatures used in the real-time PCR assays are listed in Table 2 and real-time PCR conditions are presented in Supplementary Table S1. Real-time PCR results were interpreted qualitatively as presence or absence of each target gene. A reaction was considered positive when amplification occurred above the defined threshold and was supported by an appropriate melting curve profile. Reactions with no Ct value, non-specific amplification, or values below the established limit of detection were considered negative. Borderline reactions were reported separately and were not included in the calculation of positive detection frequencies. The 16S rRNA gene assay was used as a reference marker to assess DNA amplifiability and assay sensitivity, with the lowest reliably detected standard corresponding to 10 copies/reaction. Because the study focused on qualitative screening of selected ARGs, gene-specific quantitative limits of detection were not established for all targets. The limit of detection (LOD) of the 16S rRNA gene qPCR assay was defined as the lowest quantified standard reliably detected, corresponding to 10 copies/reaction, with a mean Ct value of approximately 31.03.

### 2.5. Statistical Analysis

Descriptive statistical analysis and figures were performed using Python version 3.13.5 to summarize the phenotypic and molecular findings. For each bacterial group, the proportions of non-WT isolates and isolates positive for the targeted antimicrobial resistance genes were calculated as the number of isolates presenting the respective outcome divided by the total number of isolates tested. Results are reported as absolute frequencies (n/N) and percentages. Given the limited number of isolates and the presence of proportions close to 0% or 100%, two-sided 95% confidence intervals (95% CIs) for all proportions were calculated using the Wilson score method without continuity correction. No inferential comparisons between bacterial groups or sampling periods were performed because of the exploratory study design, the small isolate numbers, and differences in the antimicrobial agents and ARG panels examined for each bacterial group.

## 3. Results

### 3.1. Antimicrobial Susceptibility Profiles of Bacterial Isolates

**Figure 1:**
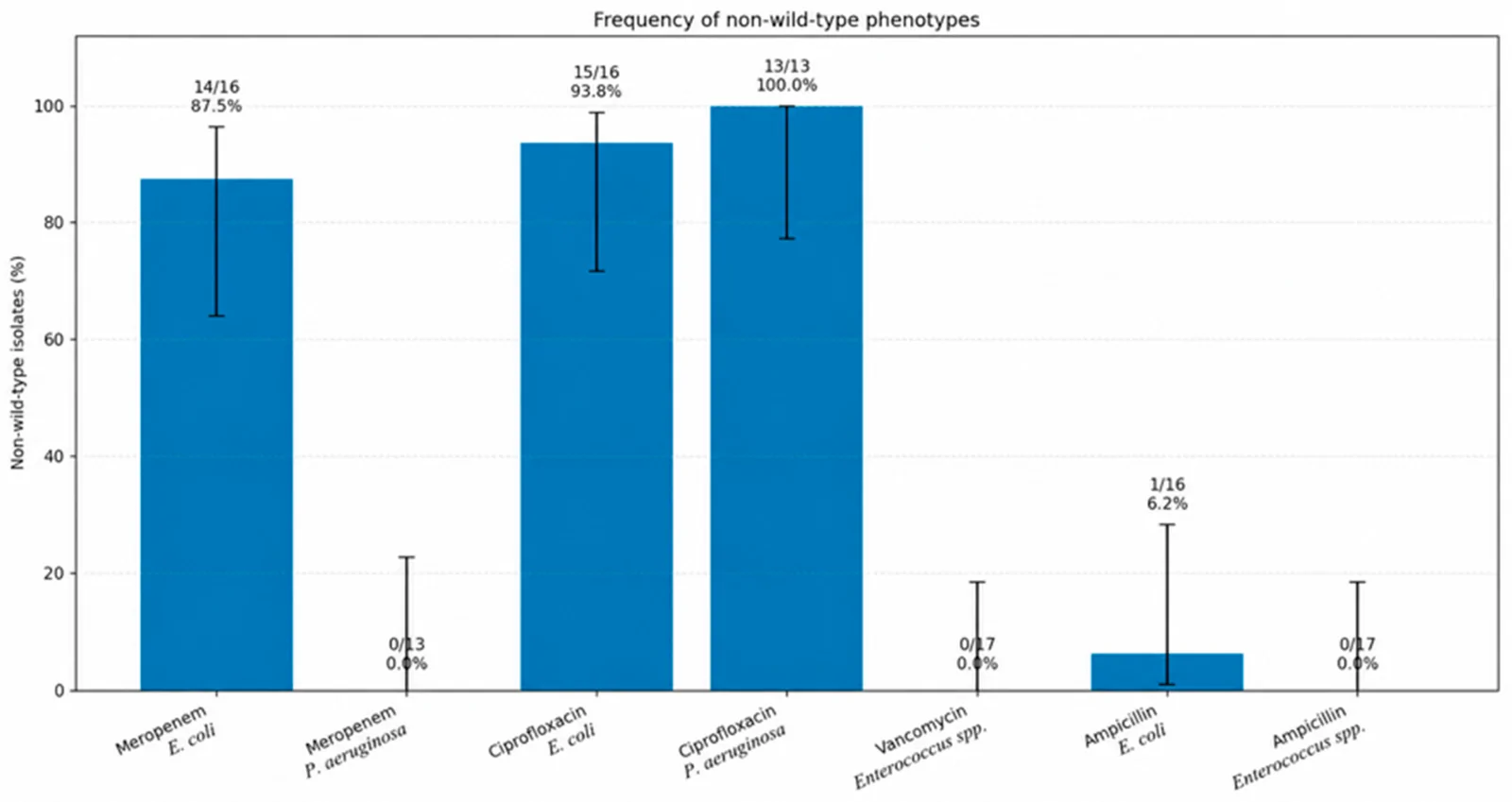
Frequency of non-wild-type (non-WT) phenotypes among bacterial isolates recovered from untreated urban wastewater, expressed as the percentage of non-WT isolates per antibiotic for each bacterial group.

A total of 16 *E. coli*, 13 *P. aeruginosa*, and 17 *Enterococcus* spp. isolates were included in the phenotypic antimicrobial susceptibility analysis. Among the *E. coli* isolates, non-WT profiles were detected in 14/16 isolates for meropenem, corresponding to 87.5% (95% CI: 64.0–96.5%), and in 15/16 isolates for ciprofloxacin, corresponding to 93.8% (95% CI: 71.7–98.9%). Ampicillin non-WT profiles were observed in 1/16 isolate, corresponding to 6.3% (95% CI: 1.1–28.3%). For *P. aeruginosa*, all 13 isolates were classified as non-WT for ciprofloxacin, corresponding to 100% (95% CI: 77.2–100.0%), whereas none were classified as non-WT for meropenem, corresponding to 0% (95% CI: 0.0–22.8%). Among the 17 *Enterococcus* spp. isolates, no non-WT profiles were observed for either vancomycin or ampicillin, corresponding to 0% (95% CI: 0.0–18.4%) for each antimicrobial agent. The antimicrobial susceptibility testing results are summarized in Table 3, while the frequencies of non-WT phenotypes are presented in Figure 1.

**Figure 2:**
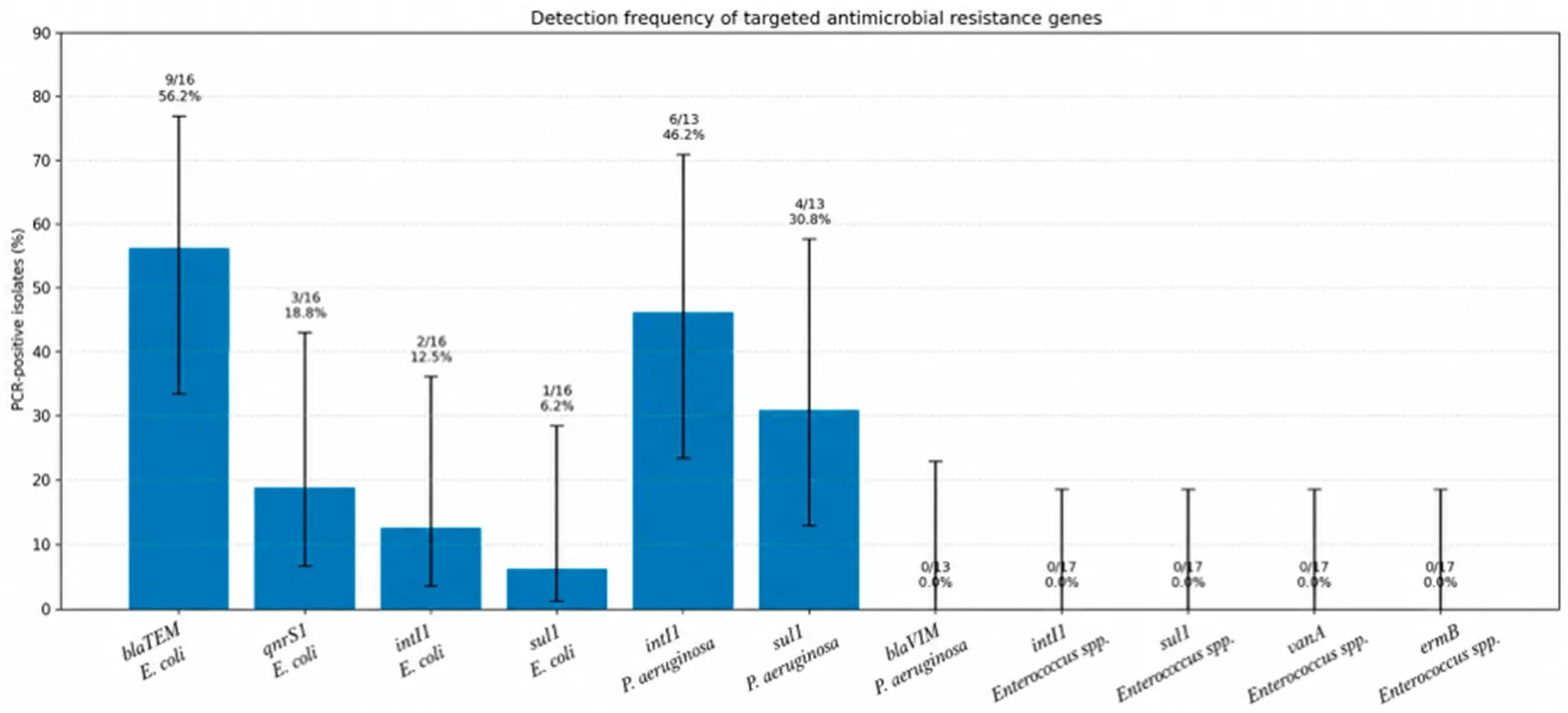
Frequency of antimicrobial resistance gene detection among bacterial isolates recovered from untreated urban wastewater, expressed as the percentage of isolates positive for each target gene by species. *E. coli* (*n* = 16) isolates were screened for *bla*TEM, *qnrS1*, *intl1* and *sul1*, *P. aeruginosa* (*n* = 13) for *intl1*, sul1 and *bla*VIM, and *Enterococcus* spp. (*n* = 17) for *intl1*, *sul1*, *vanA* and *ermB*.

Antimicrobial susceptibility results were interpreted using EUCAST ECOFFs rather than clinical breakpoints, because the isolates originated from untreated urban wastewater and were analysed for environmental AMR surveillance purposes rather than clinical decision-making. Therefore, the terms WT and non-WT were used to describe whether isolates belonged to, or deviated from, the expected WT MIC distribution. This classification indicates possible acquired resistance mechanisms or other shifts in susceptibility but should not be interpreted as clinical resistance. For example, meropenem non-WT classification in *E. coli* and ciprofloxacin non-WT classification in *P. aeruginosa* reflect interpretation against the corresponding EUCAST ECOFFs, not against clinical resistance breakpoints. The phenotypic antimicrobial susceptibility results are presented in Supplementary Table S5. Two descriptive figures were generated to visualize the findings. Figure 1 presents the frequency of non-WT phenotypes among bacterial isolates recovered from untreated urban wastewater, expressed as the percentage of non-WT isolates per antibiotic for each bacterial group and Figure 2 presents the frequency of antimicrobial resistance gene detection among bacterial isolates recovered from untreated urban wastewater, expressed as the percentage of isolates positive for each target gene.

### 3.2. Detection of Antimicrobial Resistance Genes in E. coli

Molecular analysis of the 16 *E. coli* isolates showed that *bla*TEM was the most frequently detected resistance gene, being identified in 9/16 isolates, corresponding to 56.3% (95% CI: 33.2–76.9%), with two additional isolates yielding borderline results. The plasmid-mediated quinolone resistance gene *qnrS1* was detected in 3/16 isolates, corresponding to 18.8% (95% CI: 6.6–43.0%), with one additional borderline result. The class 1 integron-associated gene *intI1* was detected in 2/16 isolates, corresponding to 12.5% (95% CI: 3.5–36.0%), whereas sul1 was detected in 1/16 isolate, corresponding to 6.3% (95% CI: 1.1–28.3%) (Figure 1). The 16S rRNA gene, included as a process and PCR control, was successfully amplified in all isolates, confirming the adequacy of the extracted DNA. These findings indicate that β-lactam resistance determinants were present in a substantial proportion of the *E. coli* isolates, accompanied by a lower frequency of plasmid-mediated quinolone, class 1 integron and sulfonamide resistance determinants (Supplementary Table S1).

### 3.3. Detection of Antimicrobial Resistance Genes in P. aeruginosa

Among the 13 *P. aeruginosa* isolates analysed, the class 1 integron-associated gene *intI1* was the most frequently detected target, being identified in 6/13 isolates, corresponding to 46.2% (95% CI: 23.2–70.9%), with one additional isolate yielding a borderline result. The sulfonamide resistance gene sul1 was detected in 4/13 isolates, corresponding to 30.8% (95% CI: 12.7–57.6%). The carbapenemase-associated gene *bla*VIM was not detected in any of the tested isolates, corresponding to 0% (95% CI: 0.0–22.8%). The *gyrA* gene, included as a process and PCR control, was successfully amplified in all isolates, confirming successful DNA extraction and template integrity. The concurrent detection of *intl1* and sul1 in a subset of isolates suggests the presence of genetic elements associated with the dissemination of antimicrobial resistance in part of the wastewater-derived population. The absence of *bla*VIM was consistent with the wild-type meropenem profiles observed among the tested *P. aeruginosa* isolates, suggesting that this carbapenemase gene was not present in the analysed collection (Supplementary Table S2).

### 3.4. Detection of Antimicrobial Resistance Genes in Enterococcus spp.

Among the 17 *Enterococcus* spp. isolates examined, none of the targeted antimicrobial resistance genes were detected. All isolates were negative for *intI1*, *sul1*, *vanA*, and *ermB*, corresponding to 0/17 isolates for each target gene, or 0% (95% CI: 0.0–18.4%). The 16S rRNA gene, included as a process and PCR control, was successfully amplified in all isolates, confirming the adequacy of the extracted DNA. These findings were consistent with the phenotypic results, as all tested *Enterococcus* spp. isolates were classified as WT for vancomycin and ampicillin (Supplementary Table S3).

## 4. Discussion

The present exploratory study provides site-specific information on selected phenotypic and genetic AMR indicators detected among bacterial isolates recovered from untreated urban wastewater in Patras. By combining culture-based antimicrobial susceptibility testing with targeted PCR screening, the study offers complementary preliminary observations on the analysed isolate collection. However, because only selected bacterial groups, antimicrobial agents, and ARGs were examined, the findings should not be interpreted as a comprehensive description of the wastewater resistome or of the community-level AMR burden. This approach is consistent with the concept of wastewater-based epidemiology as a complementary public health surveillance tool, since municipal wastewater aggregates biological material from large populations and may provide information on AMR indicators circulating beyond clinical settings [5,23,31,32,33].

Wastewater treatment plants are important interfaces between human activity, microbial pollution, antimicrobial residues and the receiving environment. Urban wastewater receives domestic, healthcare-associated and environmental inputs, creating conditions in which antibiotic-resistant bacteria, ARGs and mobile genetic elements may co-occur [23,31]. The detection of non-WT bacterial phenotypes and ARGs in untreated wastewater can thus provide useful information for community-level AMR surveillance and environmental risk assessment.

In the present study, non-WT profiles were mainly observed among Gram-negative isolates, particularly *E. coli* and *P. aeruginosa*. Among *E. coli*, 14/16 isolates were classified as non-WT for meropenem and 15/16 for ciprofloxacin, whereas only one isolate showed a non-WT profile for ampicillin. These findings suggest that reduced susceptibility to clinically relevant antibiotics was present in a substantial proportion of wastewater-derived *E. coli* isolates. *E. coli* is widely used as an indicator of faecal contamination and is also an important organism for AMR monitoring, due to its ability to acquire and disseminate resistance determinants through horizontal gene transfer [15]. Its detection in wastewater therefore provides relevant information on the possible circulation of resistant Enterobacteriaceae within the served community.

Τhe meropenem non-WT profiles observed among *E. coli* isolates and the ciprofloxacin non-WT profiles observed among *P. aeruginosa* isolates should not be interpreted as clinical resistance. In this study, isolates were classified as WT or non-WT using EUCAST ECOFFs, as they originated from untreated urban wastewater and were analysed for environmental AMR surveillance purposes. Non-WT classification indicates deviation from the expected WT MIC distribution and possible acquired resistance mechanisms, whereas clinical resistance requires interpretation according to clinical breakpoints used for therapeutic decision-making.

Molecular analysis of the *E. coli* isolates showed that *bla*TEM was the most frequently detected resistance gene, identified in 9/16 isolates, with two additional isolates showing borderline results. This finding is relevant because *bla*TEM is associated with β-lactam resistance and has been frequently reported among *E. coli* and other Enterobacteriaceae in environmental and wastewater-associated settings [5,32]. The plasmid-mediated quinolone resistance gene *qnrS1* was detected in 3/16 isolates, indicating that plasmid-mediated quinolone resistance determinants were present in part of the *E. coli* population. However, the frequency of *qnrS1* detection was lower than the frequency of ciprofloxacin non-WT phenotypes, suggesting that additional mechanisms may contribute to reduced ciprofloxacin susceptibility, including chromosomal mutations in quinolone resistance-determining regions, altered membrane permeability or efflux pump activity [34,35].

The class 1 integron-associated gene *intI1* and the sulfonamide resistance gene *sul1* were detected at lower frequencies in *E. coli.* Although these targets were not the dominant findings in this bacterial group, their detection remains important because class 1 integrons and associated resistance genes are linked to anthropogenic pollution and the mobilization of resistance determinants [36]. Consistent with our findings, an Italian study investigating WWTP effluents and receiving rivers detected *intI1*, *sul1*, *ermB*, *bla*TEM, and *qnrS*, and also reported phenotypic ciprofloxacin resistance among wastewater-associated *E. coli*, supporting the role of urban wastewater discharges in the environmental occurrence of both ARGs and antibiotic-resistant bacteria [37]. Similarly, Auguet et al. reported that *sul1* and *sul2* were the most abundant ARGs in both wastewater and sewer biofilms, while *intI1*, *qnrS*, and *bla*TEM were also detected [38]. However, direct comparison with the present detection frequencies is limited by the different analytical approaches, as these studies quantified ARGs in total wastewater and biofilm microbial communities, whereas our analysis was performed qualitatively on individual cultured isolates.

For *P. aeruginosa*, all isolates were classified as WT for meropenem, whereas all isolates were classified as non-WT for ciprofloxacin. This pattern indicates that, within the tested isolate collection, reduced susceptibility was consistently observed for ciprofloxacin but not for meropenem. *P. aeruginosa* is an opportunistic pathogen of environmental importance, with well-recognized intrinsic and acquired mechanisms. Reduced susceptibility to fluoroquinolones such as ciprofloxacin may involve several mechanisms, including mutations in quinolone resistance-determining regions, efflux pump overexpression and reduced membrane permeability [34,35].

Previous studies have shown that, in carbapenem-resistant *P. aeruginosa*, *bla*IMP and *bla*VIM may co-occur with *intI1* and *sul1*, supporting the association of carbapenemase genes with class 1 integrons and mobile resistance elements. Moreover, carbapenemase genes, particularly *bla*VIM, have been detected in isolates recovered from hospital effluent biofilms, including *Pseudomonas* spp., indicating that this determinant can persist in wastewater environments, especially where hospital-derived inputs are present [39,40]. Based on this evidence, *intI1*, *sul1*, and *bla*VIM were selected as target genes for investigation in the present study. Molecular screening of *P. aeruginosa* detected the class 1 integron-associated gene *intl1* in 6/13 isolates and the sulfonamide resistance gene *sul1* in 4/13 isolates. The detection of these genes is important from an environmental AMR perspective, as *intl1* has been proposed as a proxy marker for anthropogenic pollution and is frequently associated with genes conferring resistance to antibiotics, disinfectants and heavy metals [36]. Similarly, *sul1* is commonly detected in wastewater environments and is often associated with class 1 integrons and anthropogenic resistance gene pollution [5,36]. The presence of *intl1* and *sul1* in *P. aeruginosa* isolates indicates the occurrence of genetic elements associated with AMR dissemination in part of the wastewater-derived bacterial population.

The carbapenemase-associated gene *bla*VIM was not detected in any of the *P. aeruginosa* isolates. This finding is consistent with the phenotypic classification of all *P. aeruginosa* isolates as WT for meropenem. However, the absence of *bla*VIM does not exclude the presence of other resistance determinants or mechanisms not included in the molecular panel. Other carbapenemase genes, including *bla*NDM, *bla*KPC, *bla*IMP, and *bla*OXA-48-like, may also contribute to carbapenem resistance and were not assessed in the present study. Therefore, their potential presence cannot be excluded, and broader molecular screening or sequencing-based approaches would be required to characterize the full range of carbapenem resistance determinants in wastewater-derived isolates. Broader molecular panels or sequencing-based approaches would be required to characterize the full resistome and to identify additional genetic determinants that may be present in wastewater-derived bacterial populations [32,33].

Based on previous wastewater studies reporting the occurrence of *vanA*-positive *Enterococcus faecium* in urban and hospital wastewater, as well as the detection of *vanA* and *ermB* among wastewater-derived enterococci in several European countries [41,42,43,44] these genes were selected as relevant molecular targets in the present study. However, neither *vanA* nor *ermB* was detected among the analysed *Enterococcus* spp. isolates. This negative finding should be interpreted cautiously, as it may reflect the limited number of isolates examined rather than the complete absence of these resistance determinants.

Among *Enterococcus* spp., all tested isolates were classified as WT for both vancomycin and ampicillin. Accordingly, no phenotypic evidence of reduced susceptibility to these antibiotics was observed in the tested Enterococcus spp. isolates. Molecular analysis also showed that none of the targeted resistance genes, including *vanA*, *ermB*, *intl1* and sul1, were detected in this bacterial group. The absence of *vanA* is consistent with the wild-type vancomycin susceptibility profiles observed phenotypically. Similarly, the absence of *ermB* indicates that this specific macrolide resistance determinant was not detected in the tested isolates. However, because the isolates were not identified to species level and the molecular panel included only selected genes, further characterization would be required to fully assess the resistance potential of wastewater-derived *enterococci*.

In summary, the findings demonstrate that phenotypic and molecular AMR indicators were not uniformly distributed across the bacterial groups examined. *E. coli* showed frequent non-wild-type profiles for both meropenem and ciprofloxacin and carried several ARGs, especially *bla*TEM. *P. aeruginosa* showed a consistent ciprofloxacin non-wild-type phenotype while remaining WT for meropenem and carried *intl1* and *sul1* in a subset of isolates. In contrast, *Enterococcus* spp. isolates were phenotypically WT for the tested antibiotics and were negative for the targeted ARGs. These results highlight that culture-based phenotypic testing and targeted molecular detection provide different but complementary information.

The incomplete concordance between phenotypic and molecular findings, particularly for ciprofloxacin non-WT profiles in *E. coli* and *P. aeruginosa*, highlights the complexity of AMR characterization in environmental matrices. In several cases, the observed non-WT phenotypes were not fully explained by the specific resistance genes included in the targeted PCR panel. These may include genes not targeted by the assay, chromosomal mutations, efflux-mediated resistance, permeability changes, differential gene expression or limitations related to detection thresholds [32,34,35]. No quinolone-resistance-specific determinant or mutation analysis was included for *P. aeruginosa* in the present targeted PCR panel. Further investigation of mechanisms such as *gyrA/parC* mutations or efflux pump-related resistance was beyond the technical scope of this study, as it would have required additional validated assays or sequencing-based approaches. Whole-genome sequencing of individual isolates could provide a more comprehensive characterization of acquired resistance genes, chromosomal mutations, mobile genetic elements, and the genetic basis of the observed non-WT phenotypes. In parallel, shotgun metagenomic analysis of untreated wastewater could offer a broader, culture-independent assessment of the wastewater resistome and microbial community, including resistance determinants not captured by the targeted PCR panel. Furthermore, targeted PCR should not be interpreted as a complete substitute for phenotypic antimicrobial susceptibility testing. Instead, the two approaches should be used together to provide a broader understanding of resistance patterns in wastewater-derived bacterial isolates.

From a public health perspective, the detection of non-WT *E. coli* isolates, ciprofloxacin non-WT *P. aeruginosa* isolates and selected ARGs in untreated urban wastewater supports the value of wastewater-based monitoring as a complementary tool for AMR surveillance. Untreated wastewater can provide aggregated information on bacterial populations and resistance determinants circulating within the served community [23,33]. Wastewater-derived AMR data could complement routine clinical surveillance by providing aggregated, population-level information on resistance determinants and non-WT bacterial phenotypes circulating within a defined catchment. Integration of wastewater findings with clinical data, antimicrobial consumption records, and regional epidemiological indicators could support the identification of emerging resistance trends [45].

To our knowledge, this is the first investigation of antibiotic-resistant bacteria and selected antimicrobial resistance genes in untreated urban wastewater from the city of Patras using microbiological and molecular techniques. These findings provide a first local baseline for future wastewater-based AMR surveillance in the region. Several limitations should be considered when interpreting the findings. The study was conducted at a single wastewater treatment plant and included a limited number of isolates, which may restrict the generalizability of the results. In addition, the analysis was based on one representative colony per bacterial group and sampling event and included a restricted antimicrobial and molecular target panel. Therefore, the findings should be interpreted as preliminary, site-specific observations. Future studies should include larger numbers of isolates, longitudinal sampling, species-level confirmation of bacterial isolates and expanded molecular panels covering additional resistance genes and mechanisms. Sequencing-based approaches would also be useful to clarify the genetic basis of phenotypic resistance, investigate mobile genetic elements and explore the diversity of resistance determinants present in wastewater-derived bacteria [32,33]. Such integrated approaches could improve the interpretation of wastewater-based AMR data and strengthen its application as part of community-level and environmental AMR surveillance.

## 5. Conclusions

This study highlights the potential of untreated urban wastewater as a useful matrix for monitoring antimicrobial resistance at the community level. The detection of non-WT antimicrobial susceptibility profiles mainly among *E. coli* isolates, and for ciprofloxacin among *P. aeruginosa* isolates, indicates that wastewater can reflect the circulation of bacterial populations with reduced antimicrobial susceptibility in the served population. In contrast, *Enterococcus* spp. isolates were classified as WT for the tested antimicrobial agents, suggesting that reduced susceptibility was not observed in this bacterial group within the analysed isolate collection. Moreover, molecular analysis further confirmed the presence of selected antimicrobial resistance genes, particularly *bla*TEM in *E. coli* and *intl1* and *sul1* in *P. aeruginosa*, supporting the role of wastewater as a reservoir of resistance-associated genetic determinants. However, the partial discrepancies between phenotypic non-WT profiles and the targeted genes detected underline the complexity of antimicrobial resistance in environmental matrices. These findings suggest that targeted PCR approaches should be interpreted alongside phenotypic testing and, where possible, complemented by broader molecular methods such as expanded ARG panels or sequencing-based analyses. Ιn conclusion, wastewater-based monitoring can provide complementary information to conventional clinical surveillance and may contribute to the early detection of AMR trends within a One Health framework. Future studies should include longitudinal sampling, larger numbers of isolates, species-level confirmation, and more comprehensive molecular characterization to better understand the persistence, dissemination and public health relevance of antimicrobial resistance in urban wastewater systems.

## Figures and Tables

**Table 1:** Antibiotics and methods used for the antimicrobial susceptibility testing of the isolates and EUCAST ECOFF values for the tested antibiotics.

Bacteria	Antibiotic	Method	EUCAST ECOFF	Unit
*E. coli*	Meropenem	Etest (MIC)	0.06	mg/L
*E. coli*	Ciprofloxacin	Etest (MIC)	0.06	mg/L
*E. coli*	Ampicillin (10 μg)	Disk diffusion	13	mm
*P. aeruginosa*	Meropenem	Etest (MIC)	2	mg/L
*P. aeruginosa*	Ciprofloxacin	Etest (MIC)	0.5	mg/L
*Enterococcus* spp.	Vancomycin	Etest (MIC)	4	mg/L
*Enterococcus* spp.	Ampicillin (2 μg)	Disk diffusion	12	mm

**Table 2:** Primer sequences and annealing temperatures used for real-time PCR assays.

Genes	Primer Sequence	Annealing Temperature (°C)	Reference
*intl1*	5′-GATCGGTCGAATGCGTGT-3′ 5′-GCCTTGATGTTACCCGAGAG-3′	59	[5]
*sul1*	5′-CGCACCGGAAACATCGCTGCAC-3′ 5′- TGAAGTTCCGCCGCAAGGCTCG-3′	60	[5]
*qnrS1*	5′-GACGTGCTAACTTGCGTGAT-3′ 5′-TGGCATTGTTGGAAACTTG-3′	62	[27]
*bla*TEM	5′-TTCCTGTTTTTGCTCACCCAG-3′ 5′-CTCAAGGATCTTACCGCTGTTG-3′	60	[5]
*ermB*	5′-CCGAACACTAGGGTTGCTC-3′ 5′-ATCTGGAACATCTGTGGTATG-3′	55	[5]
*vanA*	5′-TCTGCAATAGAGATAGCCGC-3′ 5′-GGAGTAGCTATCCCAGCATT-3′	62	[28]
*bla*VIM	5′-GTTTGGTCGCATATCGCAAC-3′ 5′-AATGCGCAGCACAGGATAG-3′	58	[29]
*gyrA*	5′-AGTCCTATCTCGACTACGCGAT-3′ 5′-AGTCGACGGTTTCCTTTTCCAG-3′	60	[30]
16S rRNA gene	5′-TCCTACGGGAGGCAGCAGT-3′ 5′-ATTACCGCGGCTGCTGG-3′	60	[5]

**Table 3:** Antimicrobial susceptibility testing results and classification as WT or non-WT for bacterial isolates recovered from urban wastewater. Etest results were interpreted using MIC-based EUCAST ECOFFs, while disk diffusion results were interpreted using the corresponding zone-diameter ECOFFs. “—” indicates that the specific antimicrobial agent was not tested or was not applicable for the respective bacterial group.

Sampling Date	Microorganism	MER. Etest	CIP. Etest	VAN. Etest	Amp. 10 μg Disk	Amp. 2 μg Disk
9 June 2025	*E. coli*	non-WT	non-WT	—	WT	—
17 June 2025	*E. coli*	non-WT	non-WT	—	WT	—
25 August 2025	*E. coli*	non-WT	non-WT	—	WT	—
26 August 2025	*E. coli*	non-WT	non-WT	—	WT	—
27 August 2025	*E. coli*	non-WT	non-WT	—	WT	—
15 September 2025	*E. coli*	non-WT	non-WT	—	WT	—
17 September 2025	*E. coli*	non-WT	non-WT	—	WT	—
29 October 2025	*E. coli*	non-WT	non-WT	—	WT	—
3 November 2025	*E. coli*	non-WT	non-WT	—	WT	—
24 November 2025	*E. coli*	non-WT	non-WT	—	non-WT	—
25 November 2025	*E. coli*	non-WT	non-WT	—	WT	—
26 November 2025	*E. coli*	non-WT	non-WT	—	WT	—
1 December 2025	*E. coli*	non-WT	non-WT	—	WT	—
12 May 2026	*E. coli*	non-WT	non-WT	—	WT	—
13 May 2026	*E. coli*	WT	WT	—	WT	—
18 May 2026	*E. coli*	WT	non-WT	—	WT	—
1 September 2025	*P. aeruginosa*	WT	non-WT	—	—	—
3 September 2025	*P. aeruginosa*	WT	non-WT	—	—	—
8 September 2025	*P. aeruginosa*	WT	non-WT	—	—	—
10 September 2025	*P. aeruginosa*	WT	non-WT	—	—	—
15 September 2025	*P. aeruginosa*	WT	non-WT	—	—	—
17 September 2025	*P. aeruginosa*	WT	non-WT	—	—	—
22 October 2025	*P. aeruginosa*	WT	non-WT	—	—	—
27 October 2025	*P. aeruginosa*	WT	non-WT	—	—	—
29 October 2025	*P. aeruginosa*	WT	non-WT	—	—	—
3 November 2025	*P. aeruginosa*	WT	non-WT	—	—	—
24 November 2025	*P. aeruginosa*	WT	non-WT	—	—	—
13 May 2025	*P. aeruginosa*	WT	non-WT	—	—	—
18 May 2025	*P. aeruginosa*	WT	non-WT	—	—	—
8 September 2025	*Enterococcus* spp.	—	—	WT	—	WT
10 September 2025	*Enterococcus* spp.	—	—	WT	—	WT
15 September 2025	*Enterococcus* spp.	—	—	WT	—	WT
17 September 2025	*Enterococcus* spp.	—	—	WT	—	WT
22 October 2025	*Enterococcus* spp.	—	—	WT	—	WT
27 October 2025	*Enterococcus* spp.	—	—	WT	—	WT
29 October 2025	*Enterococcus* spp.	—	—	WT	—	WT
3 November 2025	*Enterococcus* spp.	—	—	WT	—	WT
24 November 2025	*Enterococcus* spp.	—	—	WT	—	WT
25 November 2025	*Enterococcus* spp.	—	—	WT	—	WT
1 December 2025	*Enterococcus* spp.	—	—	WT	—	WT
9 February 2026	*Enterococcus* spp.	—	—	WT	—	WT
10 February 2026	*Enterococcus* spp.	—	—	WT	—	WT
11 February 2026	*Enterococcus* spp.	—	—	WT	—	WT
12 May 2026	*Enterococcus* spp.	—	—	WT	—	WT
13 May 2026	*Enterococcus* spp.	—	—	WT	—	WT
18 May 2026	*Enterococcus* spp.	—	—	WT	—	WT

## Data Availability

The original contributions presented in this study are included in the article/Supplementary Materials. Further inquiries can be directed to the corresponding author.

## Supplementary Materials

The following supporting information can be downloaded at: https://www.mdpi.com/article/10.3390/microorganisms14081714/s1. Table S1: Real-time PCR conditions. Table S2: qPCR results for target gene detection in *E. coli* isolates from untreated urban wastewater. Table S3: qPCR results for target gene detection in *P. aeruginosa* isolates from untreated urban wastewater. Table S4: qPCR results for target gene detection in *Enterococcus* spp. isolates from untreated urban wastewater. Table S5: Complete MIC values and inhibition-zone diameters for the bacterial isolates tested.
