# Supplementary material for: Baseline Investigation of Non-Wild-Type Bacterial Indicators and Antibiotic Resistance Genes in Urban Wastewater from Patras, Greece
Source: Microorganisms. 2026 Aug 4;14(8):1714. doi: 10.3390/microorganisms14081714 (PMC13515597; doi:10.3390/microorganisms14081714)
Supplement: Supplementary file 1 [file microorganisms-14-01714-s001.zip › microorganisms-4432852-supplementary.pdf]

Supplementary Tables

Table S1: Real-time PCR conditions

Steps	Thermal Profile	Number of Cycles
Initial Denaturation	95	1
Denaturation	95	40
Extension	55-61	
Melt Curve	According to instrument recommendation	1

Table S2: qPCR results for target gene detection in *E. coli* isolates from untreated urban wastewater. Results are shown as Ct values from duplicate reactions. Yellow-highlighted cells indicate positive results, whereas light-blue-highlighted cells indicate borderline results. No Ct denotes no detectable amplification. The 16S rRNA gene was included as a bacterial reference marker.

Sampling date	*Int-1*		*Sul-1*		*qnrS1*		*blaTEM*		*16SrRNA gene*	
9/6/2025	NoCt	34,12	NoCt	NoCt	NoCt	NoCt	29,40	29,10	21,33	24,65
17/6/2025	28,22	27.57	NoCt	37,83	NoCt	NoCt	30,05	32,49	22,59	22,86
25/8/2025	33,22	31,37	NoCt	NoCt	NoCt	NoCt	29,07	29,16	29,74	24,87
26/8/2025	NoCt	37,29	NoCt	NoCt	NoCt	NoCt	24,12	23,81	24,87	24,06
27/8/2025	NoCt	32,78	NoCt	NoCt	NoCt	NoCt	29,44	29,47	27,94	26,90
15/9/2025	33,02	NoCt	NoCt	NoCt	NoCt	NoCt	31,42	31,00	25,98	27,57
17/9/2025	NoCt	NoCt	NoCt	NoCt	NoCt	NoCt	29,20	32,16	23,65	24,71
29/10/2025	31,66	32.79	NoCt	NoCt	30,25	30,08	NoCt	NoCt	28,63	27,57
3/11/2025	32,47	31.94	34,89	34,73	31.28	31,93	29,51	29,51	26,78	27,85
24/11/2025	33,26	31.65	34,31	35,13	NoCt	NoCt	28,46	29,03	25,84	26,98
25/11/2025	31,99	31.42	NoCt	34,90	NoCt	NoCt	34,69	34,56	24,98	24,78
26/11/2025	32,46	32.29	34,84	35,16	35,00	34,37	34,64	34,97	27,65	26,49
1/12/2025	32,02	32.65	35,67	35,06	NoCt	NoCt	27,14	27,01	23,85	22,75
12/5/2026	32,98	32,67	32,53	33,21	28,27	27,98	31,55	31,64	29,27	27,84
13/5/2026	33,81	32,45	36,28	34,62	30,13	31,02	31,78	NoCt	26,60	27,01
18/5/2026	22,18	22,67	28,33	28,29	24,31	24,57	23,86	28,33	22,23	23,98

Table S3: qPCR results for target gene detection in *P. aeruginosa* isolates from untreated urban wastewater. Results are shown as Ct values from duplicate reactions. Yellow-highlighted cells indicate positive results, whereas light-blue-highlighted cells indicate borderline results. No Ct denotes no detectable amplification. The *gyrA* gene was included as a bacterial reference marker.

Sampling date	***Int1***		***Sul-1***		***blaVIM***		***gyrA***	
1/9/2025	35,79	NoCt	NoCt	NoCt	NoCt	NoCt	24,45	23,88
3/9/2025	34,92	33,96	NoCt	NoCt	NoCt	NoCt	23,70	25,21
8/9/2025	NoCt	NoCt	NoCt	NoCt	37,81	36,62	22,46	24,29
10/9/2025	38,42	34,65	NoCt	NoCt	36,88	35,85	21,84	23,18
15/9/2025	NoCt	NoCt	36,71	NoCt	NoCt	NoCt	24,05	24,29
17/9/2025	19,86	19,69	NoCt	22,40	NoCt	NoCt	29,08	28,86
22/10/2025	29,46	30,33	36.31	34.53	36,79	36,83	20,24	19,81
27/10/2025	18,22	18,54	19,72	19,77	36,50	36,51	18,32	18,66
29/10/2025	30,55	30,09	33.44	35.59	NoCt	NoCt	22,48	23,20
3/11/2025	21,02	21,08	24,45	24,40	NoCt	NoCt	21,17	21,87
24/11/2025	18,31	18,34	18,86	18,52	NoCt	NoCt	19,82	21,69
13/5/2026	19,15	19,32	NoCt	NoCt	NoCt	NoCt	19,89	21,50
18/5/2026	NoCt	NoCt	NoCt	NoCt	NoCt	NoCt	23,98	23,50

Table S4: qPCR results for target gene detection in *Enterococcus spp.* isolates from untreated urban wastewater. Results are shown as Ct values from duplicate reactions. Yellow-highlighted cells indicate positive results, whereas light-blue-highlighted cells indicate borderline results. No Ct denotes no detectable amplification. The *16S rRNA* gene was included as a bacterial reference marker.

Sampling date	*Int-1*		*Sul-1*		*VanA*		*ermB*		*16SrRNA*	
8/9/2025	NoCt	NoCt	NoCt	NoCt	NoCt	NoCt	35,92	NoCt	28,73	27,45
10/9/2025	34,09	NoCt	NoCt	NoCt	NoCt	NoCt	NoCt	NoCt	24,87	27,54
15/9/2025	NoCt	NoCt	NoCt	NoCt	NoCt	NoCt	NoCt	NoCt	28,74	26,48
17/9/2025	34,65	NoCt	NoCt	NoCt	NoCt	NoCt	34,66	36,71	26,89	25,93
22/10/2025	NoCt	NoCt	NoCt	NoCt	NoCt	NoCt	NoCt	NoCt	27,57	25,89
27/10/2025	NoCt	35,68	38,19	NoCt	NoCt	NoCt	35,49	NoCt	28,65	27,46
29/10/2025	NoCt	NoCt	NoCt	NoCt	NoCt	NoCt	NoCt	35,81	28,64	26,67
3/11/2025	34,96	36.57	NoCt	NoCt	NoCt	NoCt	NoCt	36,76	28,96	28,01
24/11/2025	NoCt	35,8	NoCt	NoCt	NoCt	NoCt	36,02	NoCt	28,94	28,23
25/11/2025	NoCt	NoCt	NoCt	NoCt	NoCt	NoCt	37,24	NoCt	26,78	24,98
1/12/2025	36,86	36,18	NoCt	NoCt	NoCt	NoCt	33,8	NoCt	26,86	26,19
9/2/2026	NoCt	NoCt	34,9	35,48	NoCt	NoCt	35,16	NoCt	27,85	27,84
10/2/2026	36,21	37.04	36,4	35,7	36,03	NoCt	35,24	35,67	28,9	26,54
11/2/2026	36,58	33,94	35,46	36,12	NoCt	37,9	35,81	38,3	27,49	26,34
12/5/2026	NoCt	NoCt	NoCt	NoCt	NoCt	NoCt	NoCt	NoCt	27,56	28,35
13/5/2026	NoCt	NoCt	NoCt	NoCt	NoCt	NoCt	NoCt	NoCt	26,45	27,09
18/5/2026	NoCt	NoCt	NoCt	NoCt	NoCt	NoCt	NoCt	NoCt	28,79	27,21

Table S5: Complete MIC values and inhibition-zone diameters for the bacterial isolates tested.

Sampling Date	Microorganism	MER. Classification	MER. MIC (mg/L)	CIP. Classification	CIP. MIC (mg/L)	VAN. Classification	VAN. MIC (mg/L)	Amp. 10 µg disk Classification	Amp. zone10 µg disk (mm)	Amp. 2 µg disk Classification	Amp. zone 2 µg disk (mm)
09/06/2025	*E. coli*	non-WT	0,25	non-WT	0,12	—	—	WT	15	—	—
17/06/2025	*E. coli*	non-WT	0,38	non-WT	0,25	—	—	WT	15	—	—
25/08/2025	*E. coli*	non-WT	0,25	non-WT	0,25	—	—	WT	14	—	—
26/08/2025	*E. coli*	non-WT	0,125	non-WT	0,25	—	—	WT	14	—	—
27/08/2025	*E. coli*	non-WT	0,125	non-WT	0,12	—	—	WT	14	—	—
15/09/2025	*E. coli*	non-WT	0,125	non-WT	0,38	—	—	WT	15	—	—
17/09/2025	*E. coli*	non-WT	1	non-WT	0,25	—	—	WT	15	—	—
29/10/2025	*E. coli*	non-WT	0,38	non-WT	0,12	—	—	WT	14	—	—
03/11/2025	*E. coli*	non-WT	0,38	non-WT	0,12	—	—	WT	15	—	—
24/11/2025	*E. coli*	non-WT	0,25	non-WT	0,12	—	—	non-WT	10	—	—
25/11/2025	*E. coli*	non-WT	0,25	non-WT	0,094	—	—	WT	15	—	—
26/11/2025	*E. coli*	non-WT	0,125	non-WT	0,25	—	—	WT	15	—	—
01/12/2025	*E. coli*	non-WT	0,25	non-WT	0,38	—	—	WT	15	—	—
12/05/2026	*E. coli*	non-WT	0,125	non-WT	0,25	—	—	WT	18	—	—
13/05/2026	*E. coli*	WT	0,032	WT	0,006	—	—	WT	14	—	—
18/05/2026	*E. coli*	WT	0,032	non-WT	0,25	—	—	WT	15	—	—
01/09/2025	*P. aeruginosa*	WT	0,19	non-WT	1	—	—	—	—	—	—
03/09/2025	*P. aeruginosa*	WT	0,125	non-WT	0,75	—	—	—	—	—	—
08/09/2025	*P. aeruginosa*	WT	0,19	non-WT	0,75	—	—	—	—	—	—
10/09/2025	*P. aeruginosa*	WT	0,125	non-WT	0,75	—	—	—	—	—	—
15/09/2025	*P. aeruginosa*	WT	0,125	non-WT	0,75	—	—	—	—	—	—
17/09/2025	*P. aeruginosa*	WT	0,094	non-WT	3	—	—	—	—	—	—
22/10/2025	*P. aeruginosa*	WT	0,25	non-WT	1,5	—	—	—	—	—	—
27/10/2025	*P. aeruginosa*	WT	0,094	non-WT	1	—	—	—	—	—	—
29/10/2025	*P. aeruginosa*	WT	0,19	non-WT	1	—	—	—	—	—	—
03/11/2025	*P. aeruginosa*	WT	0,19	non-WT	1,5	—	—	—	—	—	—
24/11/2025	*P. aeruginosa*	WT	0,125	non-WT	0,75	—	—	—	—	—	—
13/05/2026	*P. aeruginosa*	WT	0,25	non-WT	0,75	—	—	—	—	—	—
18/05/2026	*P. aeruginosa*	WT	0,38	non-WT	1,5	—	—	—	—	—	—
08/09/2025	*Enterococcus spp.*	—	—	—	—	WT	0,5	—	—	WT	15
10/09/2025	*Enterococcus spp.*	—	—	—	—	WT	1	—	—	WT	30
15/09/2025	*Enterococcus spp.*	—	—	—	—	WT	1	—	—	WT	21
17/09/2025	*Enterococcus spp.*	—	—	—	—	WT	0,5	—	—	WT	20
22/10/2025	*Enterococcus spp.*	—	—	—	—	WT	0,75	—	—	WT	17
27/10/2025	*Enterococcus spp.*	—	—	—	—	WT	1	—	—	WT	15

29/10/2025	*Enterococcus spp.*	—	—	—	—	WT	0,5	—	—	WT	19
03/11/2025	*Enterococcus spp.*	—	—	—	—	WT	0,19	—	—	WT	13
24/11/2025	*Enterococcus spp.*	—	—	—	—	WT	1	—	—	WT	16
25/11/2025	*Enterococcus spp.*	—	—	—	—	WT	0,75	—	—	WT	20
01/12/2025	*Enterococcus spp.*	—	—	—	—	WT	0,75	—	—	WT	28
09/02/2026	*Enterococcus spp.*	—	—	—	—	WT	1,5	—	—	WT	13
10/02/2026	*Enterococcus spp.*	—	—	—	—	WT	0,5	—	—	WT	13
11/02/2026	*Enterococcus spp.*	—	—	—	—	WT	0,75	—	—	WT	15
12/05/2026	*Enterococcus spp.*	—	—	—	—	WT	1	—	—	WT	18
13/05/2026	*Enterococcus spp.*	—	—	—	—	WT	1,5	—	—	WT	16
18/05/2026	*Enterococcus spp.*	—	—	—	—	WT	0,38	—	—	WT	17

Note: MIC, minimum inhibitory concentration; MER, meropenem; CIP, ciprofloxacin; VAN, vancomycin; Amp., ampicillin; WT, wild type; non-WT, non-wild type; —, not tested or not applicable. MIC values above the corresponding EUCAST ECOFF were classified as non-WT, whereas inhibition-zone diameters below the corresponding ECOFF were classified as non-WT.
